# Factors Associated With Diagnostic Delays in Human Brucellosis in Tongliao City, Inner Mongolia Autonomous Region, China

**DOI:** 10.3389/fpubh.2021.648054

**Published:** 2021-10-05

**Authors:** Jingbo Zhai, Ruihao Peng, Ying Wang, Yuying Lu, Huaimin Yi, Jinling Liu, Jiahai Lu, Zeliang Chen

**Affiliations:** ^1^Innovative Institute of Zoonoses, Inner Mongolia University for Nationalities, Tongliao, China; ^2^Department of Epidemiology, School of Public Health, Sun Yat-sen University, Guangzhou, China; ^3^Plague and Brucellosis Prevention and Control Base, Chinese Center for Disease Control and Prevention, Baicheng, China; ^4^Key Laboratory of Livestock Infectious Diseases, Ministry of Education, Shenyang Agricultural University, Shenyang, China; ^5^Beijing Advanced Innovation Center for Soft Matter Science and Engineering, Beijing University of Chemical Technology, Beijing, China

**Keywords:** human brucellosis, risk factors, logistic regression analysis, age, pastoral area, diagnostic delays

## Abstract

The diagnostic delays pose a huge challenge to human brucellosis (HB), which increases the risk of chronicity and complications with a heavy disease burden. This study aimed to quantify and identify the associated factors in the diagnostic delays to its prevention, reduction, and elimination. This study analyzed risk factors associated with the diagnostic delays in a cross-sectional study with data collected from Tongliao City, Inner Mongolia Autonomous Region of China. Diagnostic delays were defined with a cutoff of 30, 60, and 90 days. In different delay groups, risk factors of diagnostic delays were analyzed by univariate analysis and modeled by multivariate logistic regression analysis. A total of 14,506 cases were collected between January 1, 2005, and December 31, 2017, of which the median diagnostic delays was 29 days [interquartile range (IQR): 14–54 days]. Logistic regression analysis indicated that the older age category was associated with longer diagnostic delays across all groups. Longer diagnostic delays increase with age among three delay groups (*p* for trend <0.001). Occupation as herdsman was associated with shorter diagnostic delays in group 1 with 30 days [adjusted odds ratio (aOR), 0.890 (95% CI 0.804–0.986)]. Diagnostic delays was shorter in patients with brucellosis who were reported in CDC in all delay groups [aOR 0.738 (95% CI 0.690–0.790), 0.539 (95% CI 0.497–0.586), and 0.559 (95% CI 0.504–0.621)]. Pastoral/agricultural area was associated with shorter diagnostic delays in group 1 with 30 days [aOR, 0.889 (95%CI 0.831–0.951)] and group 3 with 90 days [aOR, 0.806 (95%CI 0.727–0.893)]. Stratified analysis showed that the older age category was associated with an increased risk of a long delay in both genders (*p* < 0.05). The older age group-to-youth group OR increased along with increased delay time (*p* for trend <0.001). Furthermore, the pastoral/agricultural area was associated with a shorter delay in males (*p* < 0.05). Delays exist in the diagnosis of HB. We should pay great attention to the risk factors of diagnostic delays, such as older population, non-herdsman, non-pastoral/agricultural area, non-disease prevention, and control agencies. Effective measures should shorten the diagnostic delays, achieve early detection, diagnosis, and treatment, and reduce the risk of HB's chronicity, complications, and economic burden.

## Introduction

Human brucellosis (HB) is regarded as a neglected zoonotic infectious disease. *Brucella* is primarily transmitted from animals to animals or from animals to humans. There were two main routes of its transmission: first, occupational exposure, professional people contact with infected animals, and animal secretions. Second, consumer exposure, consumer consumption of contaminated products like unpasteurized milk, undercooked meat, and other products (1–3). Unfortunately, more than 500,000 annually worldwide HB cases reported as official statistics were unreported (4).

Generally, HB presents with nonspecific clinical manifestations, such as fever, sweating, fatigue, and arthritis. The broad-spectrum clinical representation may overlap with other diseases leading to diagnostic delays by misdiagnosis (5). Diagnostic delays of HB will cause the infection to change from acute to chronic (6). When HB diagnostic delays occur, unavailable timely treatment will increase the risk of chronicity and complication (7), resulting in more extended treatment, worse prognosis, and heavier financial burdens (8).

The Inner Mongolia Autonomous Region is one of the most critical areas for livestock husbandry, a historically endemic area of HB in China, accounting for approximately 40% of new cases in the country in recent years (9). In particular, Tongliao, an essential pastoral area in eastern Inner Mongolia, has known endemicity of HB with an incidence of 9.22–38.56/100 000 during 2007–2017 (10). Thus, it is necessary to focus on endemic areas of HB for disease control strategies.

Diagnostic delays in HB were multifactorial. Instead, few studies have utilized statistical models to analyze the factors associated with its diagnostic delays (11–14). Hence, it is crucial to decrease the diagnostic delays in HB by identifying relevant factors to reduce the risk of its chronicity and complications and mitigate the disease and economic burden accordingly.

## Materials and Methods

### Study Area

Tongliao is located in eastern Inner Mongolia in China, covering 58,863 km^2^, divided into eight counties with of 3.17 million population in 2019. The general outline is high in the south and north and low in the middle, with an altitude range of 120–1,400 m. It presents a continental monsoon climate, with an annual rainfall range of 300–485 mm/year and a yearly temperature of 0–6°C. Tongliao is bordered by the Liaoning province to the south, Jilin province to the northeast. Tongliao is one of the important agricultural and pastoral areas in China.

### Data Collection

Data for this cross-sectional study was obtained from the National Notifiable Disease Surveillance System (NNDSS). A total of 14,506 HB cases in Tongliao, Inner Mongolia Autonomous Region of China from January 1, 2005, to December 31, 2017, were collected through the NNDSS of China. In China, all suspected or confirmed HB cases as a class B notifiable infectious disease must be reported to the Centers for Disease Control and Prevention (CDC) (15). Basing on the “2007 Diagnostic Criteria of Brucellosis (WS269-−2019)” of the Chinese Ministry of Health, all confirmed HB cases in combination with historical epidemiology (contacting with infected animals or contaminated animal products, living in endemic areas, etc.), clinical presentation (fever, sweats, muscle pain, arthralgia, fatigue, splenomegaly, hepatomegaly, lymphadenectasis, etc.), and laboratory tests (rose bengal plate agglutination test, bacterial isolation, serum agglutination test, complement fixation test, etc.) were included in the study (10, 16). All case patients were not involved in this study. The study results were presented to the study population at the NNDSS of China through a monthly case report.

### Definitions and Category

Diagnostic delays were defined as the time interval between the onset of HB symptoms noted by the patient and its confirmed diagnosis in healthcare institutions. The first day the patient feels unwell is defined as the day of onset. The day when the patient is diagnosed as HB in the medical institution is defined as the date of confirmed diagnosis. The NNDSS used the standardized format to report the date of onset and diagnosis confirmation of HB.

The clinical stages were divided into three stages according to the course of HB: acute (patients with HB present clinical manifestations with a course of disease less than 3 months and a confirmed serological positive reaction), subacute (patients with HB present clinical manifestations range from 3 to 6 months, with confirmed positive serological reactions), or chronic (the course of the patients with HB is more than 6 months remain uncured, with signs and symptoms and confirm positive serological reactions). To adopt more stringent criteria, we used the time of the acute stage as the minimum cutoff point for the diagnostic delays. According to the different cutoff points of delay time (17), the diagnostic delay was divided into three delay groups, including delay group 1 cut off by 30 days (≤ 30 or >30), delay group 2 cut off by 60 days (≤ 60 or >60), and delay group 3 cut off by 90 days (≤ 90 or >90). With statistical significance, whether the delay time exceeds different cutoff points, different diagnostic delays were categorized into two types: the shorter (earlier) delay and the longer (later) delay.

In the confirmed cases of HB, gender was classified as male and female; age was categorized into four groups: ≤ 29 years old named age 1, 30–39 years old named age 2, 40–49 years old named age 3, and ≥50 years old named age 4; occupation was classified as herdsman and non-herdsman (farmer, worker, teacher, student, etc.); if they work for livestock husbandry or not, reporting institution was defined as CDC and non-CDC (hospital, community healthcare center, etc.) if they seek healthcare and diagnosis in CDC or not; and the area was classified as pastoral/agricultural area and non-pastoral/agricultural area if they live in downtown or not.

### Data Analysis and Statistics

Diagnostic delays were classified by three diagnostic delay time cutoff points to compare and analyze with other delay groups. Gender, age, occupation, reporting institution, and area were all included as independent variables screened by univariate analysis with the Pearson's χ^2^ test. Diagnostic delays were calculated and modeled by multivariate logistic regression model and recognized the risk factors linked to the shorter or longer diagnostic delays. crude odds ratio (crude OR), adjusted odds ratio (adjusted OR), and 95% confidence interval (95% CI) represent the strength of the association. When the value of OR/aOR > 1 showed that the factor was associated with a longer delay. Otherwise, the factor was associated with a shorter delay. A stratified multivariate logistic regression analysis by gender was conducted to examine whether the effect persisted across the strata.

Performed tests for linear trends were analyzed by entering the median value of the age category as a continuous variable in the model. The significance level was set at *p* ≤ 0.05. The database was constructed with Excel, and data were analyzed using SPSS 22.0 (IBM SPSS, Armonk, USA).

## Results

### Baseline Characteristics of Patients With HB Among Delay Groups

A total of 14,506 HB cases were included and analyzed in the study between January 1, 2005, and December 31, 2017, with 10,544 (75.0%) male and 3,962 (25.0%) female. The average age of the patients was 41.6 years (SD 12.3; rang 1–85); 2,441 (16.8%) patients were younger than 29 years, 3,684 (25.4%) patients were between 30 and 39 years, 4,536 (31.3%) patients were between 40 and 39 years, and 3,845 (26.5%) patients were older than 50 years. There were 7,469 (53.5%) patients reported by CDC. The median diagnostic delay time for all cases was 29 days (IQR: 14–54 days), 25 days (IQR: 12–44 days) for age 1 (≤29), 28 days (IQR: 14–52 days) for age 2 (30-39), 29 days (IQR: 14–56 days) for age 3 (40–49), and 30 days (IQR: 14–60 days) for age 4 (≥50) (Table 1). In three delay groups, the proportion of longer diagnostic delays in older age was higher than younger age (*p* < 0.05), and CDC was lower than other reporting institutions (*p* < 0.05). Apart from gender, occupation as herdsman and area as pastoral/agricultural were also associated with shorter diagnostic delays (*p* < 0.05) (Table 2).

**Table 1:** The characteristics of patients with human brucellosis (HB) (*N* = 14,506).

**Factors**	**Levels**	**n**	**%**
Gender	Male	10,544	25.0
	Female	3,962	75.0
Age	Age 1	2,441	16.8
	Age 2	3,684	25.4
	Age 3	4,536	31.3
	Age 4	3,845	26.5
Occupation	Herdsman	1,951	13.4
	Non-herdsman	12,555	86.6
Reporting institution	CDC	7,469	53.5
	Non-CDC	7,037	48.5
Area	Pastoral/agricultural	8,027	55.3
	Non-pastoral/agricultural	6,479	44.7
		**Median**	**IQR (25th−75th) percentile**
Diagnostic delays (All cases)		59	14–54
Diagnostic delays (Age 1)		25	12–44
Diagnostic delays (Age 2)		28	14–52
Diagnostic delays (Age 3)		29	14–56
Diagnostic delays (Age 4)		30	14–60

*Abbreviation: IQR, interquartile rang*.

**Table 2:** Univariate analysis of risk factors of the diagnostic delays in human brucellosis.

**Factors**	**Group 1**		***χ* ^2^**	***P***	**Group 2**		***χ* ^2^**	***P***	**Group 3**		***χ* ^2^**	***P***
	**Yes *n* (%)**	**No *n* (%)**			**Yes *n* (%)**	**No *n* (%)**			**Yes *n*(%)**	**NO *n* (%)**		
**Gender**												
Male	4,742 (45.0)	5,802 (55.0)			2,320 (22.0)	8,224 (78.0)			1,270 (12.0)	9,274 (88.0)		
Female	1,864 (47.0)	2,098 (53.0)	4.992	0.025	893 (22.5)	3,069 (77.5)	0.480	0.488	4,83 (12.2)	3,479 (87.8)	0.058	0.810
**Age**												
Age 1	1,006 (41.2)	1,435 (58.8)			440 (18.0)	2,001 (82.0)			232 (9.5)	2,209 (90.5)		
Age 2	1,647 (44.7)	2,037 (55.3)			783 (21.3)	2,901 (78.7)			420 (11.4)	3,264 (88.6)		
Age 3	2,087 (46.0)	2,449 (54.0)			1,040 (22.9)	3,496 (77.1)			563 (12.4)	3,973 (87.6)		
Age 4	1,866 (48.5)	1,979 (51.5)	33.731	0.000	950 (24.7)	2,895 (75.3)	41.973	0.000	538 (14.0)	3,307 (86.0)	30.546	0.000
**Occupation**												
Herdsman	783 (40.1)	1,168 (59.9)			339 (17.4)	1,612 (86.2)			196 (10.0)	1,755 (90.0)		
Non-herdsman	5,823 (46.4)	6,732 (53.6)	25.568	0.000	2,874 (22.9)	9,681 (77.1)	29.791	0.000	1,557 (12.4)	10,998 (87.6)	8.817	0.003
**Reporting institution**												
CDC	3,116 (41.7)	4,353 (58.3)			1,263 (16.9)	6,206 (83.1)			684 (9.2)	6,785 (90.8)		
Non-CDC	3,490 (49.6)	3,547 (50.4)	90.622	0.000	1,950 (27.7)	5,087 (72.3)	245.125	0.000	1,069 (15.2)	5,968 (84.8)	124.140	0.000
**Area**												
Pastoral/agricultural	3,533 (44.0)	4,494 (56.0)			1,726 (21.5)	6,301 (77.9)			886 (11.0)	7,141 (89.0)		
Non-pastoral/ agricultural	3,073 (47.4)	3,406 (52.6)	16.871	0.000	1,487 (23.0)	4,992 (77.0)	4.363	0.037	867 (13.4)	5,612 (86.6)	18.540	0.000

### Association Between Risk Factors and Diagnostic Delays in HB

Patients who were in older age category were associated with longer diagnostic delays consistent across group 1 [aOR 1.165 (95% CI 1.050–1.293), 1.193 (95% CI 1.079–1.318), 1.305 (95% CI 1.177–1.447)], group 2 [aOR 1.256 (95% CI 1.110–1.442), 1.333 (95% CI 1.176–1.511), 1.467 (95% CI 1.290–1.668)], and group 3 [aOR 1.257 (95% CI 1.060–1.489), 1.32 (95% CI 1.127–1.560), 1.506 (95% CI 1.278–1.775)]. We also observed an association for the longer diagnostic delays with increasing age among three delay groups (*p* for trend <0.01) (Table 3). Occupation as herdsman was associated with shorter diagnostic delays in group 1 [aOR, 0.890 (95% CI 0.804–0.986)]. CDC as the reporting institution was associated with shorter diagnostic delays in all delay groups [aOR 0.738 (95% CI 0.690–0.790), 0.539 (95% CI 0.497–0.586), 0.559 (95% CI 0.504–0.621)]. Pastoral/agricultural area was associated with shorter diagnostic delays in group 1 [aOR, 0.889 (95% CI 0.831–0.951)] and group 3 [aOR, 0.806 (95% CI 0.727–0.893)] (Table 4).

**Table 3:** Multiple logistic analysis of risk factors of diagnostic delays in human brucellosis.

**Groups**	**Crude OR (95% CI)**	Adjusted OR^*^ (95% CI)	***p* for trend**
Group 1			0.000
Age 1	1	1	
Age 2	1.153 (1.040–1.279)	1.165 (1.050–1.293)	
Age 3	1.216 (1.100–1.343)	1.193 (1.079–1.318)	
Age 4	1.345 (1.214–1.490)	1.305 (1.177–1.447)	
Group 2			0.000
Age 1	1	1	
Age 2	1.227 (1.078–1.398)	1.265 (1.110–1.442)	
Age 3	1.353 (1.195–1.532)	1.333 (1.176–1.511)	
Age 4	1.492 (1.315–1.694)	1.467 (1.290–1.668)	
Group 3			0.000
Age 1	1	1	
Age 2	1.225 (1.035–1.451)	1.257 (1.060–1.489)	
Age 3	1.349 (1.148–1.586)	1.326 (1.127–1.560)	
Age 4	1.549 (1.316–1.823)	1.506 (1.278–1.775)	

*Abbreviation: Crude OR, crude odds ration, Adjusted OR, adjusted odds ratio, 95% CI, 95% confidence interval*.

* *Adjusted for gender, occupation, reporting institution, and area*.

**Table 4:** Multiple logistic analysis of risk factors of diagnostic delays in human brucellosis.

**Factors**	**Group 1**		**Group 2**		**Group 3**	
	**Crude OR (95% CI)**	**Adjusted OR (95% CI)**	**Crude OR (95% CI)**	**Adjusted OR (95% CI)**	**Crude OR (95% CI)**	**Adjusted OR (95% CI)**
**Occupation** ^**a**^						
Non-herdsman	1	1	1	1	1	1
Herdsman	0.775 (0.703–0.854)	0.890 (0.804–0.986)	0.708 (0.626–0.802)	0.894 (0.785–1.019)	0.789 (0.674–0.923)	1.034 (0.877–1.219)
**Reporting institution** ^**b**^						
Non-CDC	1	1	1	1	1	1
CDC	0.728 (0.681–0.777)	0.738 (0.690–0.790)	0.531 (0.490–0.575)	0.539 (0.497–0.586)	0.563 (0.508–0.623)	0.559 (0.504–0.621)
**Area** ^**c**^						
Pastoral/agricultural	1	1	1	1	1	1
Non-pastoral/ agricultural	0.871 (0.816–0.931)	0.889 (0.831–0.951)	0.920 (0.850–0.995)	0.936 (0.863–1.016)	0.803 (0.727–0.888)	0.806 (0.727–0.893)

*Abbreviation: Crude OR, crude odds ration, Adjusted OR, adjusted odds ratio, 95% CI, 95% confidence interval*.

*a, adjusted for gender, age, reporting institution, and area; b, adjusted for gender, age, occupation, and area; c, adjusted for gender, age, occupation, and reporting institution.

### Subgroup Analysis

Among three different delay groups, the subgroup analysis stratified by gender demonstrated that the older age category was associated with longer diagnostic delays in men and women. In contrast, the women had a higher risk of longer diagnostic delays (*p* < 0.05). Moreover, it was also indicated that the association for the longer diagnostic delays is increasing with age among men and women for all delay groups (*p* for trend < 0.01) (Table 5). The subgroup analysis of area stratified by gender found that the pastoral/agricultural area was associated with shorter diagnostic delays in men in group 1 and group 3 (*p* < 0.05) (Table 6).

**Table 5:** Stratified analysis by gender of association between age and diagnostic delays in human brucellosis.

**Groups**	Adjusted OR^*^ (95% CI) among male	***p* for trend**	Adjusted OR^*^ (95% CI) among female	***p* for trend**
Group 1		0.003		0.000
Age 1	1		1	
Age 2	1.107 (0.984–1.246)		1.408 (1.129–1.755)	
Age 3	1.146 (1.022–1.285)		1.403 (1.139–1.729)	
Age 4	1.196 (1.061–1.348)		1.710 (1.385–2.112)	
Group 2		0.000		0.000
Age 1	1		1	
Age 2	1.233 (1.063–1.430)		1.397 (1.053–1.853)	
Age 3	1.294 (1.122–1.493)		1.489 (1.140–1.944)	
Age 4	1.407 (1.215–1.631)		1.687 (1.291–2.205)	
Group 3		0.000		0.003
Age 1	1		1	
Age 2	1.269 (1.047–1.538)		1211 (0.840–1.747)	
Age 3	1.299 (1.079–1.564)		1.400 (0.996–1.970)	
Age 4	1.479 (1.224–1.787)		1.592 (1.132–2.238)	

*Abbreviation: Crude OR, crude odds ration, Adjusted OR, adjusted odds ratio, 95% CI, 95% confidence interval*.

* *Adjusted for occupation, reporting institution, and area*.

**Table 6:** Stratified analysis by gender of association between area and diagnostic delays in human brucellosis.

**Groups**	Adjusted OR^*^ (95% CI) among male	Adjusted OR^*^ (95% CI) among female
Group 1		
Pastoral/agricultural	1	1
Non-pastoral/agricultural	0.861 (0.795–0.932)	0.972 (0.853–1.108)
Group 2		
Pastoral/agricultural	1	1
Non-pastoral/agricultural	0.933 (0.848–1.026)	0.954 (0.816–1.116)
Group 3		
Pastoral/agricultural	1	1
Non-pastoral/agricultural	0.755 (0.687–0.875)	0.904 (0.742–1.101)

*Abbreviation: Crude OR, crude odds ration, Adjusted OR, adjusted odds ratio, 95% CI, 95% confidence interval*.

* *Adjusted for age, occupation, and reporting institution*.

## Discussion

Human brucellosis, a neglected zoonotic infectious disease, has common diagnostic delays due to non-specific clinical signs. Its unavailable timely treatment will increase the risk of chronicity and complications, resulting in a lower cure rate and heavier financial burdens. So it is crucial to elucidate the related risk factors to shorten the delay time to prevent, control, and eliminate the disease.

The study showed that diagnostic delays were 29 days, slightly lower than other studies in other areas for 35 and 52 days (18, 19). However, diagnostic delays of HB exist in Tongliao. This study also indicated the association between age and diagnostic delays. The older the age, the longer delay among different delay groups (*p* for trend < 0.01). This result is similar to a study that reported a longer average diagnosis time of HB in older patients than younger (19). The population in the study area was dominated by herders and farmers, especially among older people. They tend to have low levels of education and knowledge acceptance, which impact the awareness rate of HB-related knowledge, which will affect the behavior of seeking medical care resulting in diagnostic delays of HB in older patients conclusively (20, 21). Meanwhile, the clinical manifestations of HB are usually nonspecific, presented in a variety of clinical symptoms, which could lead to misdiagnosis (22). For example, in the middle-aged and elderly population, HB usually overlaps by osteoarthritis and tuberculosis (23, 24). Moreover, there are difficulties and challenges in the diagnosis of HB (25). In addition, younger patients in HB mainly presented as acute cases, which indicates that they will seek medical care earlier than older patients, thereby shortening the diagnosis time (26). Thus, the association with longer diagnostic delays in older patients tends to be enhanced. So it is of practical significance to strengthen the health education of HB in focus population for the prevention and control.

The shorter diagnostic delays of HB were found in herdsman and pastoral/agricultural areas. As a common infectious disease, the history of epidemiology in HB is an essential basis for the diagnosis. At the same time, HB had occupational features, while herdsman is a high-risk occupation (27). Furthermore, Tongliao is one of the high-risk areas (10). Therefore, epidemiological evidence and the social-ecological surrounding are helpful for faster diagnosis. Moreover, we should pay attention to the diagnosis of HB in non-herdsmen and non-pastoral/agricultural areas. Meanwhile, shorter HB diagnostic delays were observed in the reported institution as CDC. This result could be due to its laboratory testing capabilities and its critical role in infectious diseases prevention and control.

In addition to prevent and combat human infections of HB, eliminating disease at its source is our ultimate goal. As a zoonotic infectious disease, the epidemiological and the One Health approach should engage in the prevention, control, and elimination of HB. Most human pathogens originate from animal reservoirs. In other words, animals are sentinels for human cases. It was economical and profitable to focus on the control of animals. First, there was feasible and effective through mass vaccination of livestock and test-and-slaughter interventions to ensure a low prevalence of animals, which are associated with morbidity in humans (28). Moreover, it was momentous to establish integrated surveillance-response systems to control human and animal disease (29). Animal disease spectrum has an important reference value for predicting the occurrence of human disease. Cross-information between different sectors may also provide meaningful epidemiological information. Furthermore, the convenient movement of animal commodities cross-border greatly increases the difficulty of disease prevention, control, and elimination. Thus, cross-border collaboration and coordination should establish among adjacent endemic countries to control diseases, such as Mongolia, Kazakhstan, and Kyrgyzstan (30–32).

Stratified analysis showed longer diagnostic delay risk for different age groups in women. It might be related to the social and family role. In some regions, the women are mainly responsible for housework, which might affect their knowledge of HB and lead to further treatment-seeking delay. Thus, it is necessary to strengthen the health education to the female in the region to improve the cognitive level of HB.

This study has its limitations. The data collected from NNDSS with limited independent variables cannot deeply analyze the other risk factors with diagnostic delays. Moreover, further delays cannot be studied in addition to diagnostic delays in HB, such as patient delays and health system delays (12). Furthermore, echinococcosis also is one of the important zoonotic diseases in this region. The joint prevention and control of brucellosis and echinococcosis will be a worthy study topic (33). The study data have good integrity, uniformity, accuracy, and timeliness. At the same time, the few missing values, duplicate values, and error values in the data have been appropriately processed to meet the requirements of statistical analysis to achieve an accurate representation of the actual situation of the disease. Diagnostic delays were calculated by the date of onset of HB, which may generate recall bias. However, this study assesses that diagnostic delays were longer in older age. At the same time, it is shorter for those patients with HB who were reported in CDC. Furthermore, it provided an essential reference for prevention, control, and elimination.

In conclusion, the diagnostic delays are presented in HB. Diagnostic delays are correlated with age: the older age, the longer delay. Moreover, the shorter diagnostic delays are associated with the occupation as a herdsman, area as pastoral/agricultural, and reporting institution as CDC. So it can be used as a significant reference for prevention and control measures to reduce diagnostic delays. At the same time, applying the epidemiological and the One Health approach to the elimination of disease at the sources is our ultimate goal.

## Data Availability Statement

The data analyzed in this study is subject to the following licenses/restrictions: The data supporting the findings of this study from the National Notifiable Disease Surveillance System (NNDSS) with the restrictions on the access to these data. These data are used under the permission of this study and therefore cannot be publicly available. However, the authors can obtain data under reasonable requirements and permission from the Plague and Brucellosis Prevention and Control Base. Requests to access these datasets should be directed to Ying Wang, 86257929@qq.com.

## Ethics Statement

The authors confirm the ethical policies of the journal, as mentioned in the journal's author guidelines. No human subjects work was undertaken in this non-experimental study. The data were obtained from annual case reports. All data were anonymized.

## Author Contributions

ZC and JLu conceived and designed the study. JZ, RP, YW, YL, and HY participated in data collection and analysis. RP and JZ interpreted the results and wrote the first draft of the manuscript. ZC, JLi, and JLu corrected the manuscript. All authors contributed to the manuscript and approved the submitted version.

## Funding

This work was supported by the State Key Program of National Natural Science of China (U1808202), NSFC International (regional) Cooperation And Exchange Program (31961143024), Major Science and Technology Projects of Inner Mongolia of China (2019ZD006), National Key Research and Development Program Projects of China (2017YFD0500305 and 2017YFD0500901), the National Key Program for Infectious Disease of China (2018ZX10101002-002), Key-Area Research and Development Program of Guangdong Province (2018B020241002), the National Science and Technology Major Project (No. 2018ZX10101002-001-001), and the Guangdong Provincial Science and Technology Project (2020B111112003 and 320 2018B020207013).

## Conflict of Interest

The authors declare that the research was conducted in the absence of any commercial or financial relationships that could be construed as a potential conflict of interest.

## Publisher's Note

All claims expressed in this article are solely those of the authors and do not necessarily represent those of their affiliated organizations, or those of the publisher, the editors and the reviewers. Any product that may be evaluated in this article, or claim that may be made by its manufacturer, is not guaranteed or endorsed by the publisher.
